# A splice site variant in *INPP5E* causes diffuse cystic renal dysplasia and hepatic fibrosis in dogs

**DOI:** 10.1371/journal.pone.0204073

**Published:** 2018-09-20

**Authors:** Kati J. Dillard, Marjo K. Hytönen, Daniel Fischer, Kimmo Tanhuanpää, Mari S. Lehti, Katri Vainio-Siukola, Anu Sironen, Marjukka Anttila

**Affiliations:** 1 Pathology Research Unit, Finnish Food Safety Authority, Evira, Helsinki, Finland; 2 Department of Veterinary Biosciences, University of Helsinki, Helsinki, Finland; 3 Research Programs Unit, Molecular Neurology, University of Helsinki, Helsinki, Finland; 4 The Folkhälsan Institute of Genetics, Helsinki, Finland; 5 Natural Resources Institute, LUKE, Jokioinen, Finland; 6 Institute of Biotechnology, University of Helsinki, Helsinki, Finland; 7 Institute of Biomedicine, University of Turku, Turku, Finland; University of Massachusetts Medical School, UNITED STATES

## Abstract

Ciliopathies presenting as inherited hepatorenal fibrocystic disorders are rare in humans and in dogs. We describe here a novel lethal ciliopathy in Norwich Terrier puppies that was diagnosed at necropsy and characterized as diffuse cystic renal disease and hepatic fibrosis. The histopathological findings were typical for cystic renal dysplasia in which the cysts were located in the straight portion of the proximal tubule, and thin descending and ascending limbs of Henle’s loop. The pedigree of the affected puppies was suggestive of an autosomal recessive inheritance and therefore, whole exome sequencing and homozygosity mapping were used for identification of the causative variant. The analyses revealed a case-specific homozygous splice donor site variant in a cilia related gene, *INPP5E*: c.1572+5G>A. Association of the variant with the defect was validated in a large cohort of Norwich Terriers with 3 cases and 480 controls, the carrier frequency being 6%. We observed that the identified variant introduces a novel splice site in *INPP5E* causing a frameshift and formation of a premature stop codon. In conclusion, our results suggest that the *INPP5E*: c.1572+5G>A variant is causal for the ciliopathy in Norwich Terriers. Therefore, genetic testing can be carried out in the future for the eradication of the disease from the breed.

## Introduction

Hepatorenal fibrocystic disorders (HRFCDs) are characterized by developmental portobiliary and renal fibrocystic abnormalities such as polycystic kidneys and congenital hepatic fibrosis. HRFCDs belong to a larger group of diseases called ciliopathies that are caused by structural or functional defects in the primary cilium. Ciliopathies are an expanding group of diseases that are clinically and genetically very heterogeneous and can manifest as a congenital developmental syndrome or as progressive single organ dysfunction. Autosomal recessive and dominant polycystic kidney diseases (ARPKD and ADPKD respectively) are the most common forms of HRFCDs in humans. Kidney and liver lesions also variably occur in other syndromic ciliopathies like Joubert, Meckel, Bardet-Biedl and Jeune syndrome [1].

To date pathogenic variants in two genes for ARPKD (OMIM #264200) and ADPKD (OMIM #173900), 33 in Joubert syndrome (OMIM #213300), 13 in Meckel syndrome (OMIM #249000), 21 in Bardet-Biedl (OMIM #209900) and 19 genes in Jeune syndrome (OMIM %208500) are reported in OMIM [2]. In these disease groups, the syndromic phenotype and the name of the disease can be caused by differing functional variants in a same gene.

In ciliopathies, the variants are located in genes that encode proteins associated with the structure or function of the primary cilium, an antenna like structure, which is present at the cell surface of almost all vertebral cell types. Primary cilia are composed of a 9+0 microtubule axoneme, which is anchored to the basal body and covered by a membrane that is a continuum of the plasma membrane [3]. The importance of this structure has become evident as increasing amount of data on its role in cellular signaling pathways and function as a sensor of extracellular environment have become available. Normal ciliary function is also crucial for organogenesis and developmental defects caused by various functional abnormalities can range from severe congenital syndromes to progressive disorders [4].

The rapid advances in sequencing technologies have been crucial in the acceleration of disease causing variant discovery and concomitant genetically overlapping phenotypes in ciliopathies have been revealed. These new technologies have also accelerated the unraveling of pathobiology and genetic background of naturally occurring inherited diseases in mammals, where dogs have become an important source in biomedical research [5–7]. HRFCDs have previously been reported only in two dog breeds. Polycystic kidneys with ductal plate malformations have been diagnosed in two litters of West Highland White (WHW) Terriers by the same dogs [8] and in two closely related Cairn Terrier litters [9]. In this study, we describe the pathology and genetic background of a congenital HRFCD in Norwich Terriers with a more severe phenotype than the previously reported cases in dogs.

## Results

### Pathology reveals diffuse cystic renal dysplasia and hepatic fibrosis

**Fig 1:**
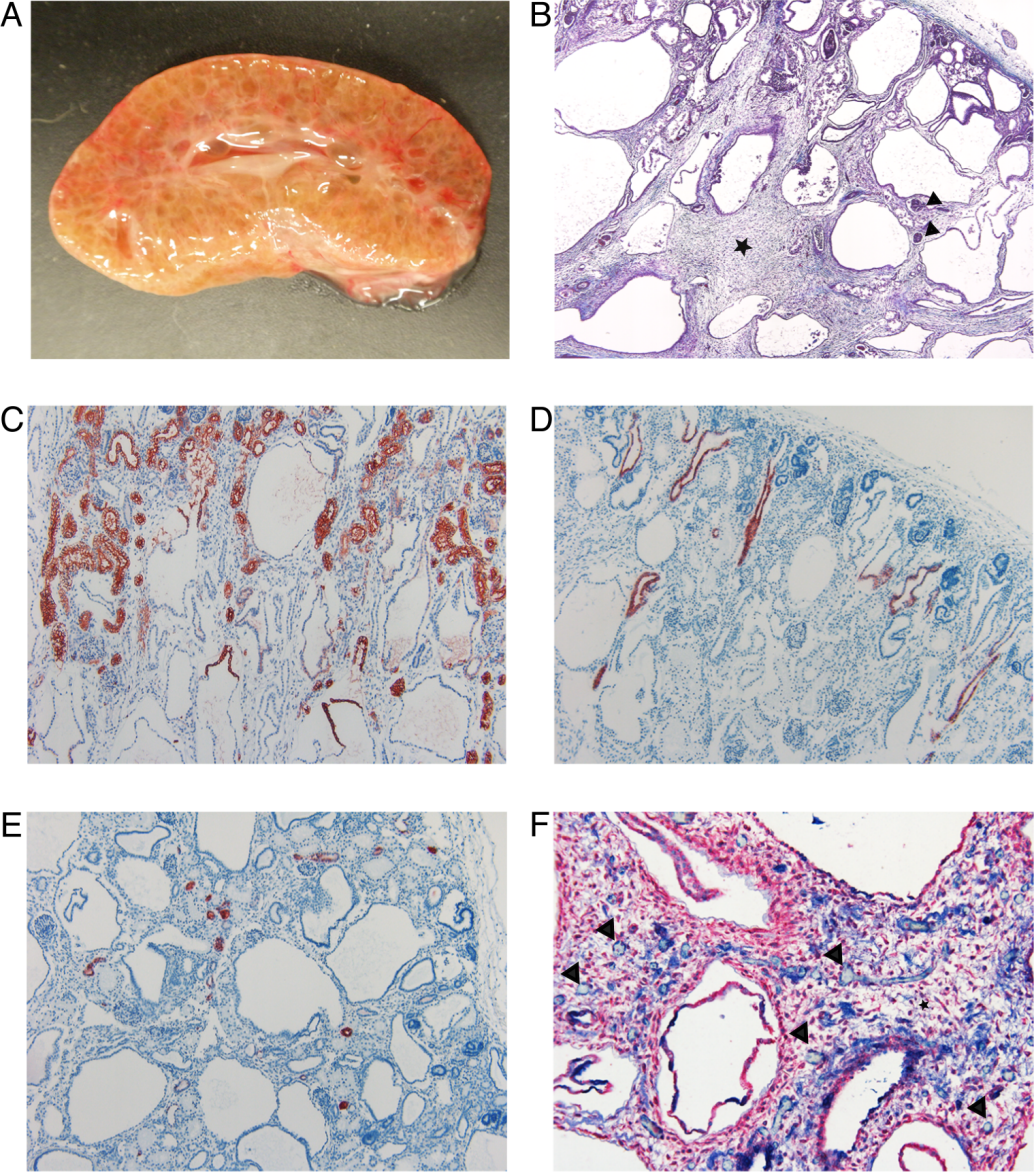
Macroscopic and histological changes of the affected kidneys. (A) Photograph of the cross-section of an affected kidney. Cortex and medulla are poorly demarcated and the parenchyma is nearly diffusely cystic. (B) Masson trichrome stain. There are immature glomeruli (black arrowheads) and normal appearing tubules in the cortex. The demarcation between the cortex and medulla is poorly defined. The tubules in the medulla are dilated and there is increased amount of loose connective tissue (black star) between the cystic tubules (4X). (C) IHC staining for AQP-1 as a marker for epithelial cells (red) of the convoluted and straight part of the proximal tubule and the thin descending limb of Henle’s loop. The convoluted portions of proximal tubules appeared morphologically normal. In the upper part of the cysts, the epithelium was positively stained indicating their origin as the straight portion of the proximal tubule and thin descending limb of Henle’s loop. The lower part of the cysts was not stained (10X). (D) IHC staining for AQP-2 as a marker for epithelial cells (red) of the collecting ducts. The collecting ducts were mainly normal. There were a few slightly dilated collecting ducts in the cortex (10X). (E) IHC staining for calbindin-D28K as a marker for epithelial cells (red) of the distal convoluted tubule. The distal convoluted tubules were morphologically normal (10X). (F) IHC double staining for α-SMA for mesenchymal cells (red) and vWF for endothelial cells (blue). The connective tissue (black star) contains scattered capillaries (black arrowheads). Only a very few capillaries are in close proximity to the tubules (20X).

Complete necropsy was performed on the three affected puppies. The abdomen was distended due to markedly enlarged kidneys. The normal reniform shape of the kidneys was somewhat retained but the kidneys were diffusely cystic. The demarcation between the cortex and medulla was not evident, the calyces were poorly formed and the renal pelvis was poorly defined (Fig 1A). The cysts were small and fairly even in size and varying between 0.5–1.5 mm in diameter. The lower urinary tract was normal in all affected puppies. There was marked variable subcutaneous edema, hydrothorax and ascites. The lungs were hypoplastic in all three puppies. There were additional variable macroscopical malformations in each puppy including cleft palate (cases 1 and 3), diaphragmatic eventration (case 2) and diaphragmatic hernia (case 3). There were no macroscopic findings in the liver.

In the histological examination, the renal parenchyma was largely replaced by cystic structures (Fig 1B). The cortex appeared thin and contained immature glomeruli and normal appearing convoluted proximal tubules, distal tubules and collecting ducts. Occasional collecting duct was mildly dilated. In addition, there were a few dysplastic glomeruli surrounded by markedly dilated Bowman’s space. There was no clear demarcation between the cortex and medulla nor between the inner and outer medulla and the medullary rays were not visible. The medulla was composed of markedly dilated tubules lined by cuboidal epithelium in the outer medulla and by flatted epithelium in the inner medulla. Some of the tubules contained hyperplastic epithelium. Between the dilated tubules, there was increased amount of loose connective tissue (mesenchyme) that contained scattered capillaries. The capillary network appeared poorly developed.

The lobular structure of the liver was normal but the portal areas were expanded and contained numerous bile ducts surrounded by connective tissue. In some portal tracts, the bile ducts were arranged circumferentially. There was no bile stasis. The findings were typical for ductal plate malformation and consistent with congenital hepatic fibrosis.

### Immunohistochemistry of the kidneys further characterizes the novel diffuse cystic dysplasia

In order to identify the affected parts of the nephron, a panel of antibodies specific for the different segments was used for immunohistochemical (IHC) staining of the kidneys. In renal diseases with severe morphological changes, it is often difficult to discern where exactly in the nephron the lesions are located without the help of immunohistochemistry. As controls for the IHC stainings, morphologically normal kidneys from adult dogs (n = 2) and puppies under 8 weeks old (n = 2) were used.

In the adult dogs, the staining for anti-aquaporin-1 (AQP-1, localization in the proximal convoluted tubule (PCT) and straight portion of proximal tubule), anti-aquaporin-2 (AQP-2, localization in the collecting ducts), anti-Tamm-Horsfall (TH, localization in the thick ascending limb of the loop of Henle and straight portion of distal tubule) and anti-calbindin- D_28K_ (CalD, localization in the distal convoluted tubule (DCT)) was identical to that reported previously [10, 11], however, the anti-α-glutathione-S-transferase (GSTA1) antibodies stained the epithelium of the straight part of proximal tubules only in one of the adult dogs and anti- KIT proto-oncogene tyrosine-protein kinase (CD117) did not stain renal cells in any of the dogs. This variability of GSTA1 expression in dogs was expected, as individual and breed differences have been previously reported [10, 11]. Also in humans, glutathione S-transferaces (GSTs) are highly polymorphic and have variable functional expression in different ethnic populations [12]. To our knowledge, anti-θ-glutathione-S-transferase (GSTT1) antibody has not been previously utilized as a renal marker in dogs. In the adult dogs, GSTT1-antibodies stained positively the epithelium of the small tubules located in inner and outer medulla, and these tubules were identified as the thin segment of Henle´s loops. CD117, anti-von Willebrand factor (vWF) and anti-cluster of differentiation 31 (CD31) antibodies were utilized for the identification of blood vessels. In the adult dogs, positive staining with CD117 was present along the capillary lumen in the inner medulla and the endothelial cells in the capillary network and the larger blood vessels were positive for vWF and CD31. The anti- α –smooth muscle actin (α-SMA) antibodies (for identification of connective tissue) stained positively the connective tissue in the portal tracts in liver and in the medulla of the kidneys as well as the wall of blood vessels.

In the 8-weeks-old control puppies the staining with AQP-1-, AQP-2-, TH-, CalD-, vWF-, CD31- and α-SMA-antibodies was similar to the adult kidneys, but CD117-, GSTA1-, and GSTT1-antibodies did not stain any cells of the nephron segments. The observed negative staining of GSTs in this age group could also be due to genetic as well as age-related differences in the canine kidney, or the expression level may be too low in young animals to be detected by immunohistochemistry [13]. Overall, the variability in the expression of GSTs makes them less useful as a marker for specific nephron segments.

In the affected puppies, the localization of the used antibodies was similar to the control puppies except for CD31. AQP-1 positive epithelium was present in morphologically normal tubules in the cortex (proximal convoluted tubules and some straight portions of proximal tubules). Epithelium in the upper part of the large cysts stained positively for AQP-1 (Fig 1C). The epithelium in the lower part of the large cysts did not stain with any of the used antibodies. The epithelium of normal and occasionally dilated collecting ducts in the cortex were positive for AQP-2 (Fig 1D). CalD and TH stained positively the epithelium of normal appearing distal convoluted tubules and straight portion of distal tubules, respectively (Fig 1E). The endothelium of capillaries and larger blood vessels stained positively with vWF whereas CD31 staining was negative in the affected puppies. There were only a few capillaries around the tubules and ducts confirming the histological finding of poorly developed capillary network. In the inner medulla, the capillaries were surrounded by α-SMA positive excessive loose connective tissue and very few capillaries were in close proximity to the tubules (Fig 1F).

In the affected puppies the staining, localization and morphology of the cysts are compatible with the straight portion of the proximal tubule, and thin descending and ascending limbs of Henle’s loop. The findings in the affected Norwich Terriers are typical for diffuse cystic dysplasia.

### Exome sequencing and homozygosity mapping revealed a variant in *INPP5E*

**Fig 2:**
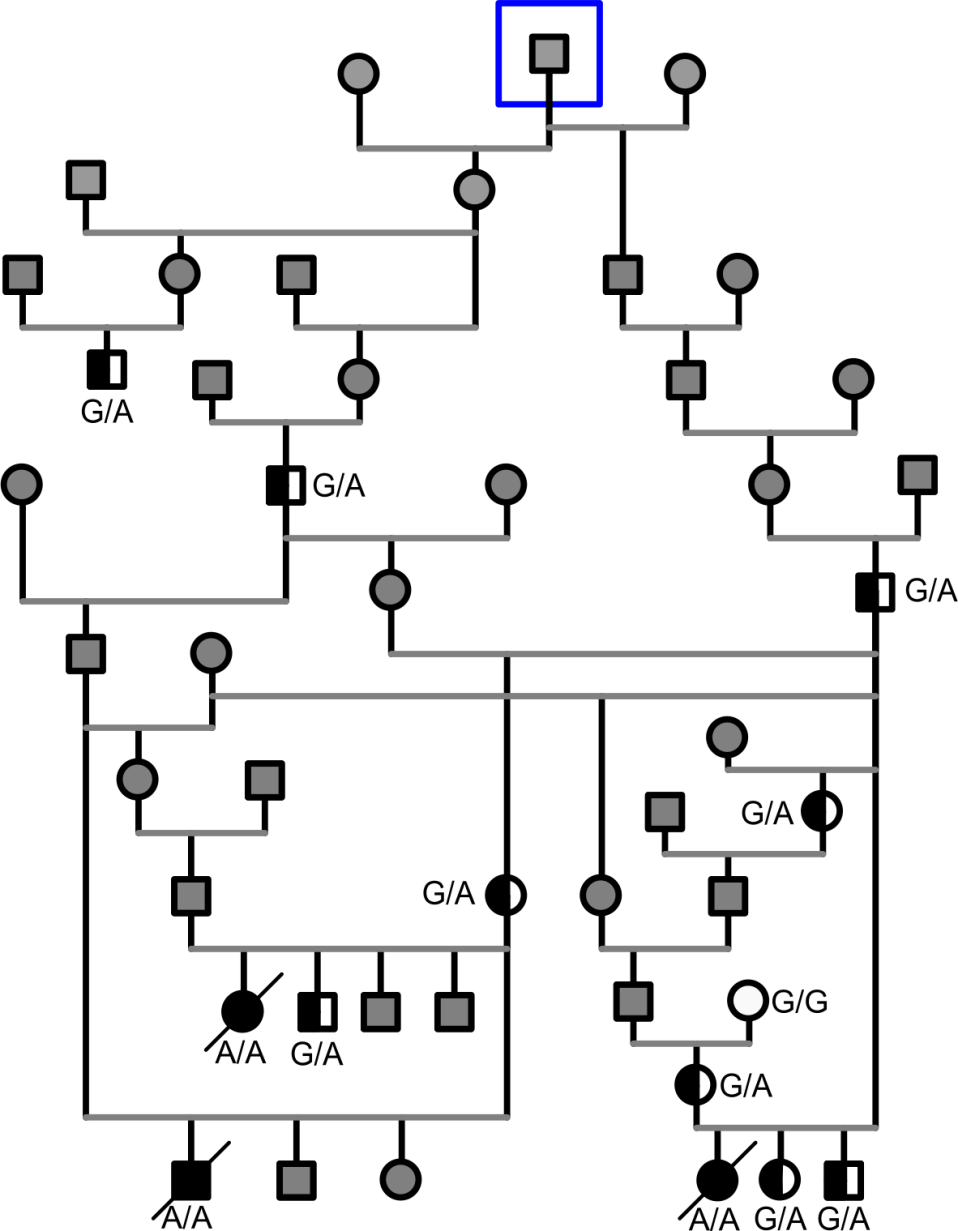
Pedigree of the affected Norwich Terrier puppies. The pedigree is suggestive of an autosomal recessive inheritance. The INPP5E genotyped dogs are denoted as affected A/A (black symbol), heterozygous G/A (half black symbol), wild-type G/G (white symbol) and gray symbol denotes the dogs that were not available for testing. Closest common ancestor to the affected puppies is surrounded with a blue square.

**Fig 3:**
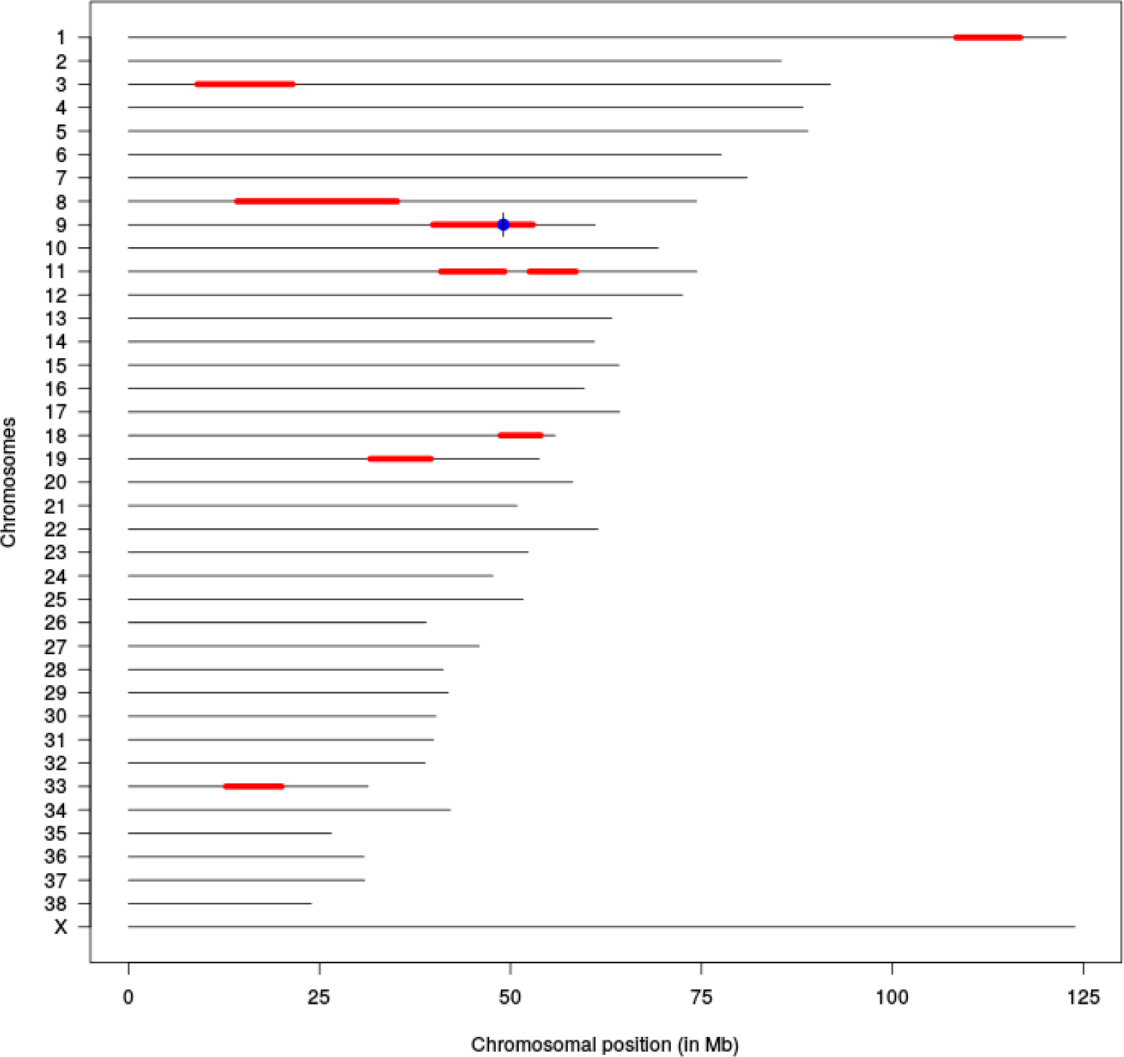
Regions of homozygosity in two affected puppies. ROH analysis revealed nine homozygous chromosomal regions (red) of over 200 continuous SNPs. The variant in the position chr9:49,069,064, INPP5E:c.1572+5G>A is indicated in blue.

The three affected puppies were from three litters with a common ancestor (Fig 2). Therefore, the disease was suspected to be inherited with an autosomal recessive manner. Whole exome sequencing (WES) was used for the identification of variants in HRFCD affected Norwich Terrier puppies. Two affected puppies, two obligate carriers and three non-affected unrelated Norwich Terrier control samples were sequenced yielding in average 72.6 million reads with 36X coverage. On average 96.8% of the total number of reads of each sample mapped to the reference genome CanFam3.1. The variant calling resulted in 214691 variants in total with 181233 SNVs and 31981 insertions and deletions across the samples (S1 Table). The variants were further analyzed by filtering according to a recessive model. This filtering yielded altogether 956 homozygous variants in the affected puppies. Since the number of remaining variants was considerably high, we decided to analyze the runs of homozygosity (ROH) in the affected animals. We performed genome-wide genotyping using Illumina´s Canine HD SNP arrays for two affected puppies, one non-affected full sibling and four close relatives and analyzed the case-specific ROH’s. This yielded nine ROH loci with more than 200 consecutive homozygous SNPs (S2 Table). The Ensembl Variant Effect Predictor (VEP) [14] was next used to predict the effects of homozygous variants within ROH regions and eight missense and eight splice-region variants in eleven different genes were identified (S4 Table). Fifteen of these variants were previously known (gene specific variant table [15]) and therefore excluded. The only remaining variant, chr9 g.49069064G>A, was at the 5´splice donor site of intron 9 in inositol polyphosphate-5-phosphatase E, *INPP5E*:c.1572+5G>A (Fig 3.) The hypothesis that this gene variant is the causative variant of HRFCD is supported by the known functional role of INPP5E in primary cilia and the malfunction of INPP5E results in ciliopathies.

### The *INPP5E* variant is rare in the Finnish Norwich Terrier population

The presence of the *INPP5E* c.1572+5G>A homozygous variant in the three affected puppies was confirmed by Sanger sequencing. For further validation, we genotyped the variant in a cohort of 480 Finnish Norwich Terriers. No other homozygous dogs were found in this cohort while 29 of the analyzed dogs were heterozygous and the association of the variant to the disease was significant (p = 8,377 x 10^−37^). The carrier frequency was 6% (29 / 483) and all carrier dogs were close relatives to the affected puppies (Fig 2). In addition, the variant was investigated in 200 dogs from 69 breeds and 3 wolves using publicly available whole genome sequencing data (S3 Table). The variant was not observed in any of the samples.

### *INPP5E* expression is altered in affected animals

**Fig 4:**
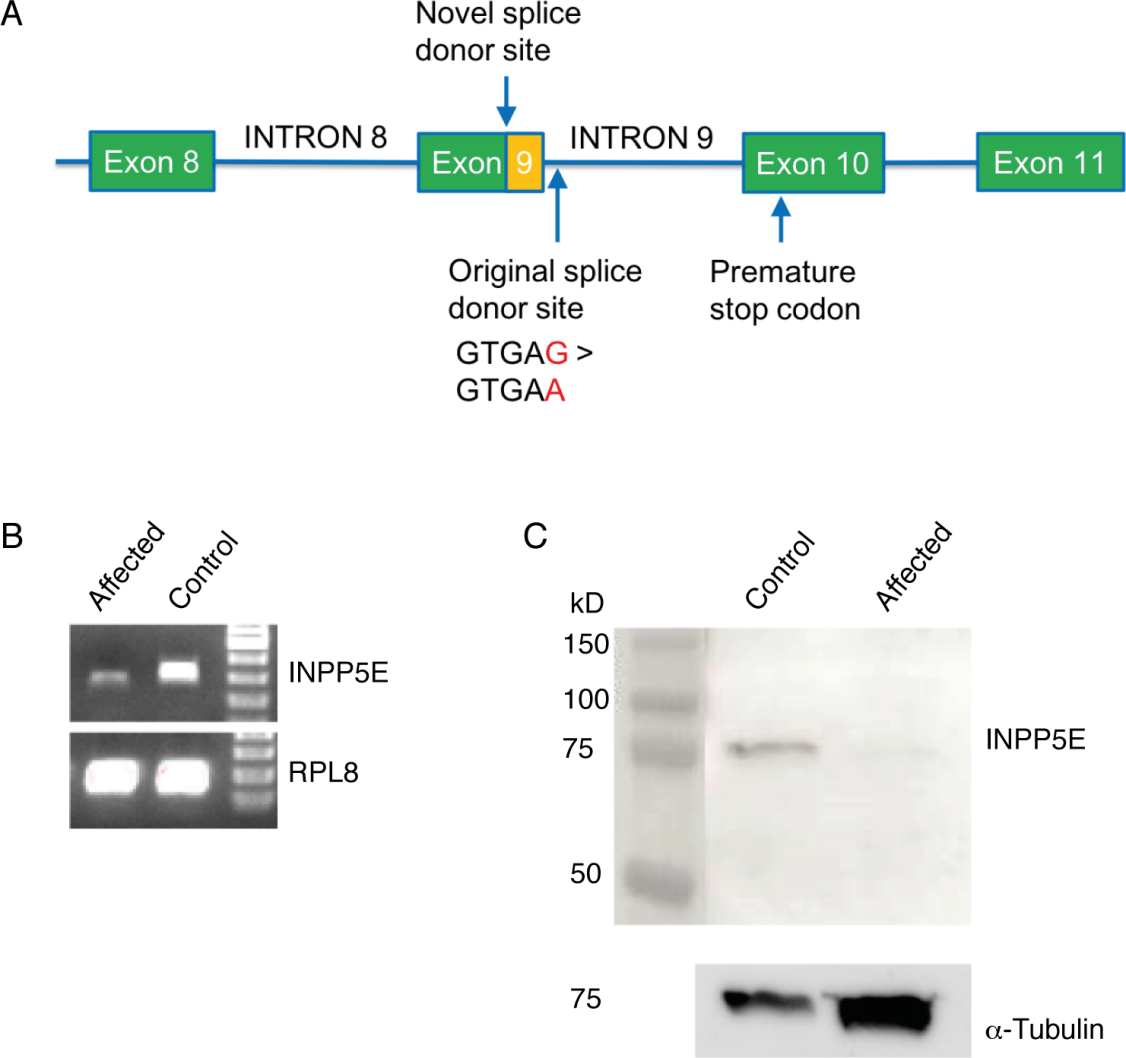
*INPP5E* mRNA and protein expression in the affected and control kidney tissue. (A) The variant causes a frameshift in INPP5E and activates a novel splice site in exon 9 deleting 50 bp from the 3´ end of exon 9 (indicated with orange box) and introducing a premature stop codon (B) A shorter mRNA product is produced in the affected kidney compared to control. (C) No wild-type INPP5E protein was detected in the Western blot of the affected kidney. α–tubulin was used as loading control.

Since *INPP5E*:c.1572+5G>A resides in a splice site, we investigated the effect on gene expression and possible functional change in the protein sequence (Fig 4A). The assessment of *INPP5E* expression by RT-PCR demonstrated a shorter mRNA product in affected puppies compared to controls (Fig 4B). Sequencing of the product revealed that the *INPP5E*:c.1572+5G>A variant in the 5´splice donor site of intron 9 caused a shift in the reading frame of *INPP5E* and activated a novel splice donor site within exon 9 deleting 50 bp from the 3´ end of exon 9 and introducing a premature stop codon (Fig 4A, S1 Text). If the abnormal transcript is translated, the protein product is predicted to be 594 aa (normal protein 613 aa) out of which the last 85 aa have an aberrant amino acid sequence (S2 Text). Subsequently, we performed Western blotting to examine the expression of the protein product. The INPP5E antibody we used targets the 530–603 amino acids of dog INPP5E, however, the predicted truncated protein contains only the first 509 amino acids of the canonical sequence. Although our Western blotting clearly demonstrates that the full-length form (72 kDa) of the INPP5E protein is not present in the affected puppies, shorter forms may be expressed. The used antibody binds amino acids 530–603 and therefore only shows the lack of the C-terminal part of the protein in the affected kidney (Fig 4C). This result supports the assumption that the premature stop codon disrupts the translation of the INPP5E protein in HRFCD affected puppies.

### Ciliogenesis in the kidney epithelial cells appears to be affected by the splice site variant INPP5E:c1572+5G>A

**Fig 5:**
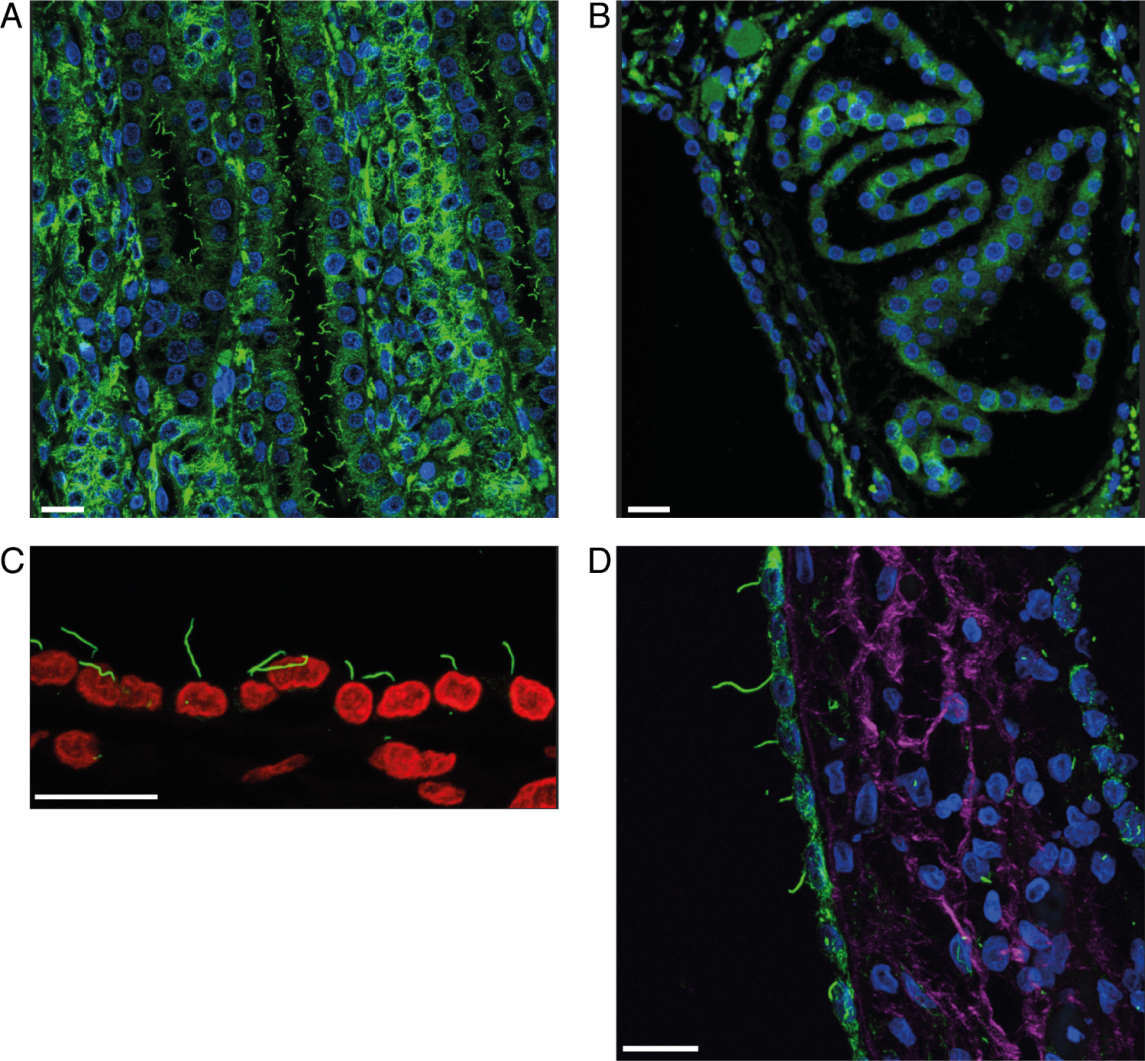
Immunofluorescence of the cilia of kidney epithelial cells in the affected and control puppies. (A) Age matched control kidney: Normal tubular epithelial cells with primary cilia. (B) Affected kidney, (case 1): Cyst lining epithelial cells with only sporadic primary cilia at the apical cell membranes. (C) Affected kidney, (Case 2): the length of the cilium varies from 2,97 to 9,25 μm in the tubular cyst lining epithelium. (D) Affected kidney, (case 2): Many of the cyst lining epithelium cells lacked a primary cilium. The length of the sparse observed cilia varied from 3,83 to 16,7 μm in the epithelial cells that had retained the cilium. For C interstitial collagen (purple) was visualized with nonlinear optical effect, second-harmonic generation (SHG). For A, B and D nucleus is shown with blue and in C with red staining, μ-tubulin was used as a marker for primary cilium (green). Scale bar 20μm.

For the characterization of the possible morphological changes caused by the *INPP5E*: c.1572+5G>A variant in the cilia of kidney tubular epithelial cells, we used immunofluorescence (α-tubulin) and confocal microscopy on FFPE kidney tissue of affected (n = 2) and control (n = 2) puppies. In the control puppies, the cilia appeared to be even in length with a normal slender morphology (Fig 5A) whereas in the affected puppies the detached tubular epithelium of the cysts mostly lacked a primary cilium (Fig 5B). In the cyst lining epithelial cells that were still anchored to the basement membrane, the cells lacked a cilium or the length varied (Fig 5C and 5D).

## Discussion

The congenital syndrome that we have characterized in the Norwich Terrier is novel both in its pathology and in its genetic background. The disease-associated variant in *INPP5E* has not been previously linked to this type of polycystic kidney disease, which is best described as diffuse cystic dysplasia. In dogs, inherited polycystic kidneys are extremely rare and have previously been reported only in three other breeds. In Bull Terriers the disease macroscopically resembles human ADPKD and the causal variant resides in the polycystin 1, transient receptor potential channel interacting (*PKD1*) gene [1, 16, 17]. In the WHW Terrier there is mild bilateral hydronephrosis without anatomical obstruction of the ureters and the renal parenchyma contains multifocal cysts. In the Cairn Terrier, the kidneys are enlarged by cysts that radiate through the cortex and medulla and the demarcation between cortex, corticomedulla and medulla is retained [8, 9]. The kidneys of the affected Norwich Terrier puppies were diffusely cystic without clear demarcation between cortical and medullary components. The unique macroscopic appearance of the kidneys differs from all previously described polycystic kidneys in dogs as well as humans and is explained by the location of the cyst in the proximal-mid section of the nephron. The closest macroscopic resemblance is to the neonatal form of ARPKD in humans, however, in this disorder the cysts are cylindrical because the cysts are in the collecting ducts [1].

The cysts in the human ARPKD, as well as in WHW- and Cairn Terriers, have also a fusiform, cylindrical shape and they are perpendicular to the cortex and medulla and extend close to the renal capsule [1, 8, 9, 18, 19]. ARPKD in humans as well as the disease in WHW Terriers affects the distal nephron and the cysts originate from the collecting ducts [8, 20]. In the Norwich Terrier, the cysts are most prominent in the medulla with few smaller cysts present in the cortex. Other distinguishing features in the Norwich Terrier are a poorly developed capillary network around the tubules, hyperplasia of tubular epithelium and the presence of increased amount of mesenchyme. Using the previously established [10, 11] IHC panel for specific nephron segments of the dog kidney, we were able to identify the location of the cysts in the Norwich puppies. The cysts originated from the straight portion of the proximal tubule and thin descending and ascending limbs of Henle´s loop. In comparison, the cysts in human ADPKD have variable origin and they have been shown to originate from both the proximal and distal nephron as well as from collecting ducts. During fetal development in human ARPKD, a transient phase of cystic dilatation of the proximal tubules has been reported, while the majority of the cysts were in the collecting ducts [20]. Similar finding of early phase of small, multifocal and transient cysts in the proximal tubules has also been reported in murine models of the polycystic kidney disease [21, 22]. The dilation of the proximal tubular segments in these cases may represent a developmental phenomenon or a transient consequence of the cystic process affecting the collecting ducts [20]. The cystic dilatation limited to the proximal-mid portion of the nephron in Norwich Terrier puppies differs from all previously described polycystic kidney diseases.

It is possible that the severity of the disease in the affected Norwich Terrier puppies may be explained by the localization of the cysts to the straight portion of the proximal tubule, and thin descending and ascending limbs of Henle´s loop. In the proximal tubule, the electrolytes and nutrients are reabsorbed from the urine, whereas the descending and ascending limbs of Henle´s loop are responsible for creating the extracellular tonicity gradient and concentration of the urine. The exact mechanisms involved in abnormal lung development in polycystic kidney disease in utero are still not well understood. There are several postulations how this could occur. One explanation is that the enlarged kidneys mechanistically compress the diaphragm, reducing the thoracic space and in this way inhibit the normal development of the lungs [23]. Another postulation is that due to decreased urine production less amniotic fluid is produced with less trophic factors that are needed for normal lung development [24]. It is possible that both these mechanisms are involved in the severe cases of human ARPKD patients and affected Norwich Terriers as research on the possible interaction of renal disease causing genetic variants and lung development is still pending.

Ductal plate abnormalities in human HRFCDs are characterized by developmental portobiliary defects, most often as congenital hepatic fibrosis [25]. In the affected Norwich Terrier puppies the histology of the liver was compatible to human cases of congenital hepatic fibrosis as well as what has been reported in dogs as a single organ lesion [26, 27]. In human HRFCDs as well as in our affected puppies, congenital hepatic fibrosis was subclinical at the time of presentation and debilitating disease was caused by renal insufficiency. Congenital hepatic fibrosis typically causes clinical symptoms later in life both in humans as well as in dogs [27, 28].

We have found a novel disease-associated variant in the *INPP5E* gene in dog that encodes a 72 kDa protein which belongs to 5-phosphatase class of enzymes that are wildly expressed and take part in the regulation of many cellular processes including embryonic development [29]. In cells, INPP5E localizes to the primary cilium where it is involved in maintaining the normal ciliary membrane phosphoinositide content and distribution [30]. In humans, variants in *INPP5E* are linked to recessively inherited ciliopathies, Joubert Syndrome 1 (JBTS1) and clinically a very different syndrome Mental retardation, Truncal obesity, Retinal dystrophy and Micropenis (MORM) [31, 32]. The hallmark lesions in JBTS1 are structural anomalies of the brain i.e. the lack of decussation of the superior cerebellar peduncles that can be visualized as the “molar tooth sign” in MRI imaging. The brains of the affected Norwich Terriers were macroscopically normal, however, small changes in the cerebellar peduncles might have been missed since the brains were routinely dissected perpendicularly to the cranio-caudal axis. The canine cerebellum is located caudally to the cerebrum, which is in contrast to the human brain where the cerebellum is ventral to the cerebrum, thus in MRI the “molar tooth sign” would not be present in a dog. Ocular, renal and hepatic lesions in JBTS1 patients are less common [33, 34]. In an *Inpp5e* knockout mouse model, the mice died soon after birth with an extreme phenotype with multiple anomalies such as anencephaly or exencephaly, bilateral anophthalmos, multiple kidney cysts, postaxial hexadactyly, bifid sternum and delayed ossification of metacarpals and phalanxes. In these mice, majority of the cysts (84%) were in the distal nephron while 14% were located in the proximal nephron. The cyst location was determined by IHC staining for AQP1 and AQP2. There were no hepatic abnormalities in these mice [32].

In 2009, Bielas *et al* [31] showed that variants in *INPP5E* cause defects in the primary cilium signaling and stability. The specialized ciliary membrane differs in composition from the cell membrane due to the presence of a diffusion barrier at the ciliary transition zone. INPP5E maintains the specific distribution of phosphatidylinositol 4-phosphate (PtdIns(4)P) at the ciliary membrane and phosphatidylinositol 4,5-bisphosphate (PtdIns(4,5)P_2)_ at the ciliary base [30]. Hedgehog signaling is directed by INPP5E as it keeps PtdIns(4,5)P_2_ at normal low level by limiting the signaling inhibitors tubby like protein 3 (TULP3) and G protein-coupled receptor 161 (GPR161), which are localized to the cilium [35, 36]. In the absence of INPP5E the levels of PtdIns(4,5)P_2_ and TULP3 with its binding molecules GPR161 and Intraflagellar Transport Complex-A increase in the cilium. The buildup of these molecules restricts the normal ciliary transduction of Hedgehog signaling [30, 36] and the bulges in the ciliary shaft and tip seen in the SEM and TEM of the renal cyst-lining epithelium of *Inpp5e* knockout mice [32] may be changes relating to the buildup. In a recent study, Hardee *et al* [34] showed that JBTS1 patient fibroblasts with a missense *INPP5E* variant had fewer and shorter cilia compared to controls. Furthermore, in recent reports [37, 38] the importance of INPP5E has been linked to the initiation of ciliogenesis as well as the reversal of abnormal ciliogenesis by phosphatidylinositol (3,4,5)-triphosphate (PtdIns(3,4,5)P_3_) inhibition in *Inpp5e* zebrafish morphants. Similar mechanism can be expected to be affected by the *INPP5E* variant in dogs. Our α-tubulin immunofluorescence study on the kidney tissue of the affected puppies suggests a problem in ciliogenesis and cilia length control. Unfortunately at the time of necropsy, we were unable to obtain tissue samples for EM or fibroblasts for cell culture, thus the exact morphology of the cilia remains elusive in the affected puppies.

Human INPP5E has an N-terminal proline rich domain, Class I and II SH3 binding sites, inositol polyphosphate phosphatase catalytic domain (IPPc) and a C- terminal CAAX motif. In JBTS1, nonsense and missense variants are clustered within or flanking the IPPc domain. In MORM syndrome a variant causes the loss of a highly conserved CAAX motif [32, 33]. The variant identified in our study resides in the IPPc domain of dog INPP5E similar to the human JBTS1 patients, but splice site variants have not been reported in humans so far [33, 34]. The phenotypic difference between humans and Norwich Terriers with *INPP5E* variants could be due to the differences in the genetic background or depletion of different functional sites or isoforms of the protein.

In this study, we have described the detailed pathology and genetic background of renal cystic dysplasia and congenital hepatic fibrosis that cause neonatal mortality in Norwich Terriers. The genetic and functional studies confirmed the *INPP5E*: c.1572+5G>A as a causal variant for HRFCD in Norwich Terriers. The three cases and the heterozygous carriers belonged to an isolated family of Finnish Norwich Terriers. The common ancestor that links this family to the overall breed population in Finland could be traced back to 15 generations. To our knowledge, this is the first report of a genetically defined syndromic ciliopathy in a dog breed that results in neonatal mortality. The variant described here can be utilized as a preventive gene test when breeding Norwich Terriers.

## Materials and methods

### Ethics statement

All tissue samples used in this study were collected at necropsy from animals that were voluntarily sent for pathological examination by the breeders. These animals had died or had been humanely euthanized due to a disease. Blood samples from immediate family members of the affected Norwich Terrier puppies were collected with the owners’ consent and with an approval by Regional State Administrative Agency for Southern Finland ESAVI/7248/04.10.07/2014. Buccal swab samples from Norwich Terriers were voluntarily collected and submitted to the study by their owners.

### Study cohort

The three affected puppies (cases 1, 2 and 3) were from three different but closely related litters. Cases 1 (male) and 2 (bitch) were delivered at term by cesarean section. They both failed to thrive and died at two days of age. The delivery of case 3 (bitch) was normal and at term. The puppy was born alive but died within minutes after birth due to severe respiratory failure. A full necropsy was performed on the three affected puppies.

Tissue samples were collected from the three affected puppies during necropsy and blood samples were obtained from their three parents and one grandparent for DNA extraction. We collected buccal swab samples from 271 adult dogs and utilized previously collected tissue samples from 209 necropsied Norwich Terrier puppies. These samples were utilized in the variant segregation and allele frequency analysis in the Norwich Terrier population. Furthermore, publicly available whole genome sequencing data from 200 dogs representing 69 breeds and three wolves was utilized for the screening of the variant (S3 Table).

The Finnish Kennel Club´s pedigree registry KoiraNet [39] was used to obtain the pedigree information and individual dog owners provided additional information that was missing in this registry. GenoPro genealogy software [40] was used to establish a combined pedigree of the affected puppies.

### Tissue samples for histology

Samples from all major organs and macroscopically abnormal tissues were collected during necropsy. For histology, samples were fixed in 10% buffered formalin, routinely processed and embedded in paraffin. 4 μm sections were cut and stained with Hematoxylin and Eosin (HE). Special stains Masson Trichrome (MTRI) for fibrosis and Hall´s stain for bile were used.

### Immunohistochemistry

For immunohistochemistry, the following primary antibodies and dilutions were used. Anti-aquaporin-1 (AQP-1) rabbit polyclonal antibody (Merck, AB2291) diluted at 1:1000; Anti-aquaporin-2 (AQP-2) rabbit polyclonal antibody (Sigma-Aldrich, A7310) diluted at 1:500; Anti-α-glutathione-S-transferase (GSTA1) rat polyclonal antibody diluted at 1:500 (Biorbyt, orb157401) diluted at 1:100; Anti-θ-glutathione-S-transferase (GSTT1) rabbit polyclonal antibody (Thermo Fisher Scientific, PA5-43186) diluted at 1:1000; Anti-Tamm-Horsfall (TH) glycoprotein sheep polyclonal antibody (Merck, AB733) diluted at 1:250; Anti-calbindin-D_28K_ (CalD) mouse monoclonal (Sigma-Aldrich, SAB4200543) diluted at 1:1000; Anti-α-smooth muscle actin (α-SMA) mouse monoclonal antibody (Sigma-Aldrich, A5228) diluted at 1:200; Anti-CD31/PECAM-1 rabbit polyclonal antibody (Thermo Fisher Scientific, RB-10333-P1) diluted at 1:25; Anti-CD117 rabbit polyclonal antibody (Dako, A4502) diluted at 1:100; and Anti-von Willebrand Factor (vWF) rabbit polyclonal antibody (Dako, A0082) diluted at 1:100.

Formalin-fixed, paraffin embedded tissue sections were deparaffinized in UltraClear (J.T. Baker) and rehydrated in graded ethanol series. Heat induced antigen retrieval was used for all except α-SMA and vWF. The sections were heated in a microwave for 20 minutes in citrate buffer (pH 6). For CD117 Dako target retrieval solution (pH 9) was used. No antigen retrieval was used with α-SMA. For vWF the sections were incubated with proteinase K for 10 minutes at 37 ^o^C. All primary antibodies were incubated for 60 minutes at room temperature, except AQP-1, GSTA1 and vWF antibodies which were incubated 14 hours at 4 ^o^C. Ultravision ONE HRP polymer and AEC chromogen detection system kits (Thermo Fisher Scientific, TL-015-PHJ) were used according to manufacturer´s instructions with all but sheep anti-Tamm-Horsfall antibody for which anti-sheep IgG rabbit secondary antibody HRP (Thermo Fischer Scientific, 31480) was used. The sections were counterstained with hematoxylin and mounted with aqueous mounting medium.

### Immunofluorescence

For immunofluorescence, formalin-fixed, paraffin-embedded kidney tissues were cut into 8 μm thick sections. The samples were first deparaffinised in xylene or Ultraclear (J.T. Baker) and rehydrated in graded ethanol series. For heat induced antigen retrieval, the sections were pretreated with pH 6 citrate buffer in a microwave oven for 20 minutes or in 10 mM Citrate buffer + 0,05% Tween 20 in a pressure cooker. After a blocking step with goat normal serum for five minutes or with blocking buffer (10% NDS, 3% BSA in 0,01% Triton in PBS) for 1h, the sections were incubated overnight at +4° with monoclonal mouse anti-acetylated-α-tubulin [6-11B-1] (Abcam, ab24610, 1:200 or Thermo Fisher Scientific, MS-581-P0, 1:1000). Some sections were treated with image-iT®FX-signal enhancer (Thermo Fisher Scientific) for 30 minutes at RT and then incubated for 1 hour with Alexa Fluor® 488 goat anti-Mouse IgG (H+L) secondary antibody (Thermo Fisher Scientific, Molecular probes) diluted at 1:200 at 37°C or 1:500 at RT, respectively. Nuclei were counterstained using DAPI (Sigma-Aldrich, D9542) and the slides were mounted using ProLong Diamond Antifade Mountant (Life Technologies, P36970) before imaging. Prior to mounting the slides with ProLong® Gold Antifade reagent with DAPI (Thermo Fisher Scientific) some of the sections were counterstained with Eriochrome Black solution (Sigma-Aldrich) for 30 minutes at RT.

Immunofluorescence images were obtained using Leica TCS SP5 AOBS MP SMD confocal microscope with HCX APO 63xNA 1.3 glycerol immersion objective and LAS AF 2.7 software. Acetylated-α-tubulin images were obtained by using z-stack and the projections were rendered with Bitplane Imaris 7.6.5. Nonlinear optical effect, second-harmonic generation (SHG), was used to visualize collagen.

### DNA extraction

Genomic DNA from peripheral EDTA whole blood samples was extracted using QIAamp® DNA Mini Kit (Qiagen). For fresh frozen tissue we used DNeasy® Blood and Tissue Kit (Qiagen) and for formalin-fixed paraffin-embedded tissue AllPrep® DNA/RNA FFPE Kit (Qiagen). Buccal cells were collected with buccal swabs (Eurotubo®, Deltalab) and gDNA was extracted with Gentra® Puragene Buccal Cell Kit and QIAamp® DNA Mini Kit (both from Qiagen). Manufacturer´s protocols were followed in all extractions. The quantity and quality of DNA was estimated by spectrophotometric measurement (Nanodrop 1000, Thermo Fisher Scientific).

### Exome sequencing and bioinformatics analysis

Genomic DNA from two affected puppies (cases 2 and 3), dam of case 2, sire of case 3 and three control Norwich Terriers was sent to Otogenetics Corporation (Norrcross, GA, USA) for whole- exome sequencing. Standard Illumina library preparation (Illumina, USA) with quality control was performed and the coding sequences were captured with Agilent´s All Exon Canine Capture Kit (Agilent, CA, USA). This exon capture kit is a 54Mb design based on canine reference genome CanFam2 and the kit covers the UCSC tracks for Ensembl and Refseq, including human protein alignments and spliced ESTs that lie outside of Ensembl annotated gene regions. The library preparation and capture were performed according to the manufacturer´s instructions. The sequencing was performed using Illumina HiSeq2500 with 100-bp paired-end reads.

The data was aligned to CanFam3.1 reference (UCSC) and transferred to Classic DNAnexus [41] for further analysis and storage. The Classic DNAnexus exome pipeline included quality control and variant calling. The DNAnexus pipeline for variant calling [42] utilizes a probabilistic model of variation that takes into account the similarity of the sample to the reference genome (*P(H|G)*) and the possible sequencing errors (*P(R|H)*) (*H* is the probability of the genotype, *G* is the reference genome and *R* is the observed reads). Thus the probability of the genotype can be computed (*P*(*H*|*G*,*R*) = *P*(*H*|*G*) x *P*(*R*|*H*) / *P*(*R*|*G*)) and further utilized to choose the most likely genotype H* (H* = *argmax*_*H*_ {*P*(*H*|*G*,*R*) } = *argmax*_*H*_ {*P*(*H*|*G*) x *P*(*R*|*H*) }). This variant calling pipeline takes into consideration all possible genotypes with two or more reads. Assuming a recessive mode of inheritance, two affected puppies as homozygous, two obligate carriers as heterozygous or with a missing variant and controls as normal, heterozygous or with a missing variant as genotypes, were further filtered using Golden Helix ® (Bozeman, MT, USA) SNP & Variation Suite software with call rate >0.2 and MAF >0.05. In addition, the Ensembl Variant Effect Predictor (VEP) [14] that utilizes the CanFam3.1.78 annotation was used to examine the functional consequences of the variants.

The normal and affected INPP5E protein and *INPP5E* nucleotide sequences were aligned with EMBL-EBI Pairwise Sequence alignment tool [43]. Ensembl annotation of the *INPP5E* gene (ENSCAFG00000019664; ENSCAFT00000031269.3) has been used all over the study.

### SNP genotyping and homozygosity mapping

A genome-wide genotyping was performed on two affected, one non-affected full sibling and four close relatives by using Illumina's CanineHD BeadChip (Illumina, CA, USA) containing 173,662 SNP markers. The genotyping was performed by GeneSeek (Neogen, NE, USA) and the loci of the SNP markers were transformed to the CanFam3.1. reference genome using the UCSC lift-over tool [44], prior to analysis. Candidate regions were determined by analyzing the homozygosity at each loci by using R 3.2 [45]. First, monomorphic loci across the whole study population as well as loci with missing data were removed from the analysis. The set of candidate loci was then limited to the set of SNPs that were homozygous for the cases. Then, the loci of the candidate SNPs were merged to candidate regions by combining sets of consecutive candidate SNPs. To prioritize the candidate regions, the MAF of the carriers within each candidate region was determined and a priority value for each region was calculated as 2*MAF_r * length_r / max (length_r*) where MAF_r is the MAF of carriers within region r, length_r is the length of the region r in kB and max (length_r*) is the maximum length across all candidate regions. Regions with a priority value larger 0.15 were then considered further. Regions with more than 200 consecutive candidate loci were selected as candidate regions for the defect. The genotype data is available for further use upon request.

### PCR and Sanger sequencing

For Sanger sequencing, DNA fragments were amplified with *INPP5E* gene-specific primers (forward GCTCACCCAGGAAATGAAGA (exon 8), reverse CCCAGCAGTCTCAGAGAGGT (in intron 9)). The specificity of PCR amplicons was confirmed on agarose gel and 5-20ng of the PCR product was purified with Exonuclease I (10u, Thermo Fischer Scientific) at 37°C for 15 min. Purified PCR product was used for sequencing amplification with the gene-specific primers and BigDye Terminator v3.1 Cycle Sequencing Kit (Thermo Fischer Scientific). Prior to sequencing, the amplicons were precipitated with ethanol and diluted in formamide. The sequencing reaction was conducted in both directions with the same primers that were used for amplification. Sequencing was performed on a MegaBace 500 capillary DNA sequencer (Amersham Biosciences). The data was analyzed using the Variant Reporter v1.0 program (Applied Biosystems) and Sequencer 5.2.3 (Gene Codes Corporation).

### Analysis of RNA expression

Tissue samples were collected to RNAlater® (Thermo Fischer Scientific) at necropsy and stored at -20°C until extraction. RNA was extracted from kidney tissue of one affected and one unaffected Norwich Terrier stillborn control puppy. For analysis of gene expression with RT-PCR, RNA was extracted by using RNeasy Midi kit (Qiagen). Total RNA was reverse-transcribed with oligo T primers and an RT-PCR kit (ImProm-II Reverse Transcription System; Promega) according to the manufacturer’s instructions. Synthesized cDNA was diluted to 20 ng/μl prior to amplification using *INPP5E* specific primers (forward CGATGGGGTGTTCTGGTTTG (in the beginning of exon 8), reverse CCAGGGCAGGAAGAATACCT (approx. in the middle of exon 10)). The housekeeping gene RPL8 (forward GTCCGGTTCAAAGAAGGTCA, reverse GGATGCTCCACAGGATTCAT) was used as reference gene to control the equal amount of RNA in each sample. Expression of gene fragments was assessed by gel electrophoresis.

### Western blot

Kidney tissue from an affected and control puppy were homogenized using UltraTurrax homogenizator in lysis buffer [50 mM Tris–HCl pH 8.0, 170 mM NaCl, 1% Triton X-100, 5 mM EDTA, 1 mM DTT and protease inhibitors (Complete mini; Roche diagnostic)] and incubated on ice for 20 min. After centrifugation at 13000 rpm for 20 min at +4°C, 100 μg of total protein was loaded into 12% Mini-PROTEAN ® TGX Precast Gels (Bio-Rad). The gel was run at 90 V for 2 hours and electroblotted on methanol activated Hybond membrane. The membrane was first blocked with 5% non-fat milk in PBS with 0,1% Triton X-100 (PBST) for 1 hour at RT. Primary antibodies INPP5E (1:200, Biorbyt, orb184295) and α-tubulin (1:4000 NeoMarkers, MS-581-P) were diluted in 1% non-fat milk in PBST and incubated overnight at +4°C. After washes with PBST, membranes were incubated with HRP-conjugated secondary antibody [(1:3000, HRP linked anti-rabbit IgG (GE Healthcare Life Sciences)] for 1 hour at RT. Membranes were developed with ECL Plus western blotting detection system (Amersham Pharmacia) and visualized using LAS4000 (FujiFilm). Similar method has been previously used for analyses of protein expression in the testis [46]

## Supporting information

S1 Text Pairwise alignment normal and affected INPP5E coding sequence transcript. Deletion of 50 bp in the affected sequence designated in yellow and premature stop codon in red.
(DOCX)

S2 Text Alignment of normal and affected INPP5E protein sequence. The aberrant sequence in the affected is designated in yellow.
(DOCX)

S1 Table Summary of exome sequencing and variant calling. (DOCX)

S2 Table ROH loci specific for cases. (XLSX)

S3 Table Breeds and accession numbers in genome sequencing data set. (XLSX)

S4 Table Case specific variants in ROH regions. (XLSX)
