# Supplementary material for: A splice site variant in INPP5E causes diffuse cystic renal dysplasia and hepatic fibrosis in dogs
Source: PLoS One. 2018 Sep 20;13(9):e0204073. doi: 10.1371/journal.pone.0204073 (PMC6147468; doi:10.1371/journal.pone.0204073)
Supplement: S1 Text — Deletion of 50 bp in the affected sequence designated in yellow and premature stop codon in red. (DOCX) [file pone.0204073.s001.docx]

**S1 Text. Pairwise alignment normal and affected *INPP5E* coding sequence transcript.** Deletion of 50 bp in the affected sequence designated in yellow and premature stop codon in red.

INPP5E-201 1 ATGCTCAAGGGACAGCTCCCAAGCACAGGCAAAGACGTCGCCCCCCACCT 50

||||||||||||||||||||||||||||||||||||||||||||||||||

Affected 1 ATGCTCAAGGGACAGCTCCCAAGCACAGGCAAAGACGTCGCCCCCCACCT 50

INPP5E-201 51 GGGGCCCCCGCCTACCAACCACGGTCAGGATCCCGAGAGGACCCCGGTGC 100

||||||||||||||||||||||||||||||||||||||||||||||||||

Affected 51 GGGGCCCCCGCCTACCAACCACGGTCAGGATCCCGAGAGGACCCCGGTGC 100

INPP5E-201 101 TCCCTCTGACCTTTCCGGCACAAATCAGCAACGAGGACCCAGAAGCTAAG 150

||||||||||||||||||||||||||||||||||||||||||||||||||

Affected 101 TCCCTCTGACCTTTCCGGCACAAATCAGCAACGAGGACCCAGAAGCTAAG 150

INPP5E-201 151 GCAAAGCCCTTCACCCCAAAGCCCCCGCCGCAGCCCAGGCTGGAGCGGGC 200

||||||||||||||||||||||||||||||||||||||||||||||||||

Affected 151 GCAAAGCCCTTCACCCCAAAGCCCCCGCCGCAGCCCAGGCTGGAGCGGGC 200

INPP5E-201 201 TCTGTCACTGGACGAGAAAGCGTGGAGGCGGCGGCGTTTTCGGACCAGCC 250

||||||||||||||||||||||||||||||||||||||||||||||||||

Affected 201 TCTGTCACTGGACGAGAAAGCGTGGAGGCGGCGGCGTTTTCGGACCAGCC 250

INPP5E-201 251 CGGAGGACCTGGCAGCGGGCAGTGGGGCCGGTGGCTCCAGGGGCTCCTTG 300

||||||||||||||||||||||||||||||||||||||||||||||||||

Affected 251 CGGAGGACCTGGCAGCGGGCAGTGGGGCCGGTGGCTCCAGGGGCTCCTTG 300

INPP5E-201 301 CAGGACGAGGTCCCCAGGCCTCCCGGCCCCCCTGGGTCCCCACCCTGCTT 350

||||||||||||||||||||||||||||||||||||||||||||||||||

Affected 301 CAGGACGAGGTCCCCAGGCCTCCCGGCCCCCCTGGGTCCCCACCCTGCTT 350

INPP5E-201 351 GAGTACCTCCCTGCAGGAGATCCCCACATCCCGCCGGGCCCAGGACAGCG 400

||||||||||||||||||||||||||||||||||||||||||||||||||

Affected 351 GAGTACCTCCCTGCAGGAGATCCCCACATCCCGCCGGGCCCAGGACAGCG 400

INPP5E-201 401 CAGGTGGCAGCCCCTCCTCGTGGGGCCACTGTATCTCCGGAATGATCAGC 450

||||||||||||||||||||||||||||||||||||||||||||||||||

Affected 401 CAGGTGGCAGCCCCTCCTCGTGGGGCCACTGTATCTCCGGAATGATCAGC 450

INPP5E-201 451 ACTTCCCTGGACCTCCTGCACCGGGATGGGGCCGTGGCTGGGACCAGCGT 500

||||||||||||||||||||||||||||||||||||||||||||||||||

Affected 451 ACTTCCCTGGACCTCCTGCACCGGGATGGGGCCGTGGCTGGGACCAGCGT 500

INPP5E-201 501 CAGGCTGCCTCCTGTGGACCCCAAGGTGGCCCCCGAGTCCCTGCGGCCCA 550

||||||||||||||||||||||||||||||||||||||||||||||||||

Affected 501 CAGGCTGCCTCCTGTGGACCCCAAGGTGGCCCCCGAGTCCCTGCGGCCCA 550

INPP5E-201 551 CACACAGAGTGGACTCAGGTCCGGTTGATGGCAAGCCCCACCTACAGAGC 600

||||||||||||||||||||||||||||||||||||||||||||||||||

Affected 551 CACACAGAGTGGACTCAGGTCCGGTTGATGGCAAGCCCCACCTACAGAGC 600

INPP5E-201 601 AGACTGTTCCGCGCCCACAGCAGCCTGGGCCCTGGCCGGCCCCCAAGCCC 650

||||||||||||||||||||||||||||||||||||||||||||||||||

Affected 601 AGACTGTTCCGCGCCCACAGCAGCCTGGGCCCTGGCCGGCCCCCAAGCCC 650

INPP5E-201 651 CCTCGTCTGTGAGGCCAGATCCTCCTTCAGCCTCCTGGCCCCCATCCGCG 700

||||||||||||||||||||||||||||||||||||||||||||||||||

Affected 651 CCTCGTCTGTGAGGCCAGATCCTCCTTCAGCCTCCTGGCCCCCATCCGCG 700

INPP5E-201 701 CCAAGGACGTCCGGAGCAGGAGCTACCTCGAGGGGAGTCTCCTGGCAAGT 750

||||||||||||||||||||||||||||||||||||||||||||||||||

Affected 701 CCAAGGACGTCCGGAGCAGGAGCTACCTCGAGGGGAGTCTCCTGGCAAGT 750

INPP5E-201 751 GGGGCCCTGATGGGGGCGGAGGAACTGGCCCGGTACTTCCCAGACCGCAA 800

||||||||||||||||||||||||||||||||||||||||||||||||||

Affected 751 GGGGCCCTGATGGGGGCGGAGGAACTGGCCCGGTACTTCCCAGACCGCAA 800

INPP5E-201 801 CCTGGCCCTCTTCGTGGCCACCTGGAACATGCAAGGTCAGAAGGAGCTGC 850

||||||||||||||||||||||||||||||||||||||||||||||||||

Affected 801 CCTGGCCCTCTTCGTGGCCACCTGGAACATGCAAGGTCAGAAGGAGCTGC 850

INPP5E-201 851 CCCCAAACCTGGATGAGCTCCTGCTGCCTGCTGAGGCTGACTACGCCCAG 900

||||||||||||||||||||||||||||||||||||||||||||||||||

Affected 851 CCCCAAACCTGGATGAGCTCCTGCTGCCTGCTGAGGCTGACTACGCCCAG 900

INPP5E-201 901 GACCTGTATGTCATCGGGGTCCAGGAGGGCTGCTCCGACAGGCGGGAGTG 950

||||||||||||||||||||||||||||||||||||||||||||||||||

Affected 901 GACCTGTATGTCATCGGGGTCCAGGAGGGCTGCTCCGACAGGCGGGAGTG 950

INPP5E-201 951 GGAGACGCGCCTGCAGGAGACGCTGGGTCCCCGCTACGTCACGCTGTACT 1000

||||||||||||||||||||||||||||||||||||||||||||||||||

Affected 951 GGAGACGCGCCTGCAGGAGACGCTGGGTCCCCGCTACGTCACGCTGTACT 1000

INPP5E-201 1001 CGGTAGCCCATGGGGCACTCTACATGTCTGTGCTCATCCGCAGGGACCTC 1050

||||||||||||||||||||||||||||||||||||||||||||||||||

Affected 1001 CGGTAGCCCATGGGGCACTCTACATGTCTGTGCTCATCCGCAGGGACCTC 1050

INPP5E-201 1051 ATCTGGTTCTGCTCAGAGGTGGAGAGCTCCACGGTGACCACGCGCATCGT 1100

||||||||||||||||||||||||||||||||||||||||||||||||||

Affected 1051 ATCTGGTTCTGCTCAGAGGTGGAGAGCTCCACGGTGACCACGCGCATCGT 1100

INPP5E-201 1101 ATCTCACATCAAGACCAAGGGGGCCCTGGGCGTCAGCTTCACTTTCTTCG 1150

||||||||||||||||||||||||||||||||||||||||||||||||||

Affected 1101 ATCTCACATCAAGACCAAGGGGGCCCTGGGCGTCAGCTTCACTTTCTTCG 1150

INPP5E-201 1151 GCACCTCTTTTCTCTTCATCACATCCCATTTCACCTCTGGAGACGGGAAG 1200

||||||||||||||||||||||||||||||||||||||||||||||||||

Affected 1151 GCACCTCTTTTCTCTTCATCACATCCCATTTCACCTCTGGAGACGGGAAG 1200

INPP5E-201 1201 GTGAGCGAGAGGCTGCTGGACTATAGCAGAACCATCCAGGGCCTGGCCCT 1250

||||||||||||||||||||||||||||||||||||||||||||||||||

Affected 1201 GTGAGCGAGAGGCTGCTGGACTATAGCAGAACCATCCAGGGCCTGGCCCT 1250

INPP5E-201 1251 GCCCAAGAGTGTGCCTGACACCAGCCCCTACCGCTCCGATGCTGCGGATG 1300

||||||||||||||||||||||||||||||||||||||||||||||||||

Affected 1251 GCCCAAGAGTGTGCCTGACACCAGCCCCTACCGCTCCGATGCTGCGGATG 1300

INPP5E-201 1301 TCACCACCCGGTTCGATGGGGTGTTCTGGTTTGGAGACTTCAACTTCCGT 1350

||||||||||||||||||||||||||||||||||||||||||||||||||

Affected 1301 TCACCACCCGGTTCGATGGGGTGTTCTGGTTTGGAGACTTCAACTTCCGT 1350

INPP5E-201 1351 CTGAGTGGTGGGCGCGTGGCCGTGGAGGCCATCCTGAAGCAGGACCTGGT 1400

||||||||||||||||||||||||||||||||||||||||||||||||||

Affected 1351 CTGAGTGGTGGGCGCGTGGCCGTGGAGGCCATCCTGAAGCAGGACCTGGT 1400

INPP5E-201 1401 AGAGAAGGTGTCTACTCTGCTCCAGCATGACCAGCTCACCCAGGAAATGA 1450

||||||||||||||||||||||||||||||||||||||||||||||||||

Affected 1401 AGAGAAGGTGTCTACTCTGCTCCAGCATGACCAGCTCACCCAGGAAATGA 1450

INPP5E-201 1451 AGAAAGGGTCCATCTTCAAGGGCTTCCAGGAGCCAGACATCCATTTTCTT 1500

||||||||||||||||||||||||||||||||||||||||||||||||||

Affected 1451 AGAAAGGGTCCATCTTCAAGGGCTTCCAGGAGCCAGACATCCATTTTCTT 1500

INPP5E-201 1501 CCATCCTACAAGTTCGACATTGGTAAGGACTCCTACGACACCACCTCCAA 1550

|||||||||||||||||||||

Affected 1501 CCATCCTACAAGTTCGACATT----------------------------- 1521

INPP5E-201 1551 GCAAAGGACCCCATCCTACACGGACCGGGTCATGTACAGAAGCCGCCACA 1600

|||||||||||||||||||||||||||||

Affected 1522 ---------------------GGACCGGGTCATGTACAGAAGCCGCCACA 1550

INPP5E-201 1601 AGGGTGACATCTGTCCAGTCAGGTATTCTTCCTGCCCTGGCATCAAGACG 1650

||||||||||||||||||||||||||||||||||||||||||||||||||

Affected 1551 AGGGTGACATCTGTCCAGTCAGGTATTCTTCCTGCCCTGGCATCAAGACG 1600

INPP5E-201 1651 TCTGACCACCGCCCCGTGTACGGCCTGTTCCGGGTCAAAGTGAGGCCGGG 1700

||||||||||||||||||||||||||||||||||||||||||||||||||

Affected 1601 TCTGACCACCGCCCCGTGTACGGCCTGTTCCGGGTCAAAGTGAGGCCGGG 1650

INPP5E-201 1701 GAGAGACAACATCCCGCTAGCTGCTGGCAAGTTTGACCGAGAGCTGTACT 1750

||||||||||||||||||||||||||||||||||||||||||||||||||

Affected 1651 GAGAGACAACATCCCGCTAGCTGCTGGCAAGTTTGACCGAGAGCTGTACT 1700

INPP5E-201 1751 TGATAGGAATCAAAAGACGGATTTCCAAAGAAATCCAGAGACAGCAAGCA 1800

||||||||||||||||||||||||||||||||||||||||||||||||||

Affected 1701 TGATAGGAATCAAAAGACGGATTTCCAAAGAAATCCAGAGACAGCAAGCA 1750

INPP5E-201 1801 CTGAAAAATCAGCACTCGAGTACGATTTGTACCGTGTCTTGA 1842

||||||||||||||||||||||||||||||||||||||||||

Affected 1751 CTGAAAAATCAGCACTCGAGTACGATTTGTACCGTGTCTTGA 1792
