# Supplementary material for: A splice site variant in INPP5E causes diffuse cystic renal dysplasia and hepatic fibrosis in dogs
Source: PLoS One. 2018 Sep 20;13(9):e0204073. doi: 10.1371/journal.pone.0204073 (PMC6147468; doi:10.1371/journal.pone.0204073)
Supplement: S2 Text — The aberrant sequence in the affected is designated in yellow. (DOCX) [file pone.0204073.s002.docx]

**S2 Text. Alignment of normal and affected INPP5E protein sequence.** The aberrant sequence in the affected is designated in yellow.

INPP5E-201 1 MLKGQLPSTGKDVAPHLGPPPTNHGQDPERTPVLPLTFPAQISNEDPEAK 50

||||||||||||||||||||||||||||||||||||||||||||||||||

Affected 1 MLKGQLPSTGKDVAPHLGPPPTNHGQDPERTPVLPLTFPAQISNEDPEAK 50

INPP5E-201 51 AKPFTPKPPPQPRLERALSLDEKAWRRRRFRTSPEDLAAGSGAGGSRGSL 100

||||||||||||||||||||||||||||||||||||||||||||||||||

Affected 51 AKPFTPKPPPQPRLERALSLDEKAWRRRRFRTSPEDLAAGSGAGGSRGSL 100

INPP5E-201 101 QDEVPRPPGPPGSPPCLSTSLQEIPTSRRAQDSAGGSPSSWGHCISGMIS 150

||||||||||||||||||||||||||||||||||||||||||||||||||

Affected 101 QDEVPRPPGPPGSPPCLSTSLQEIPTSRRAQDSAGGSPSSWGHCISGMIS 150

INPP5E-201 151 TSLDLLHRDGAVAGTSVRLPPVDPKVAPESLRPTHRVDSGPVDGKPHLQS 200

||||||||||||||||||||||||||||||||||||||||||||||||||

Affected 151 TSLDLLHRDGAVAGTSVRLPPVDPKVAPESLRPTHRVDSGPVDGKPHLQS 200

INPP5E-201 201 RLFRAHSSLGPGRPPSPLVCEARSSFSLLAPIRAKDVRSRSYLEGSLLAS 250

||||||||||||||||||||||||||||||||||||||||||||||||||

Affected 201 RLFRAHSSLGPGRPPSPLVCEARSSFSLLAPIRAKDVRSRSYLEGSLLAS 250

INPP5E-201 251 GALMGAEELARYFPDRNLALFVATWNMQGQKELPPNLDELLLPAEADYAQ 300

||||||||||||||||||||||||||||||||||||||||||||||||||

Affected 251 GALMGAEELARYFPDRNLALFVATWNMQGQKELPPNLDELLLPAEADYAQ 300

INPP5E-201 301 DLYVIGVQEGCSDRREWETRLQETLGPRYVTLYSVAHGALYMSVLIRRDL 350

||||||||||||||||||||||||||||||||||||||||||||||||||

Affected 301 DLYVIGVQEGCSDRREWETRLQETLGPRYVTLYSVAHGALYMSVLIRRDL 350

INPP5E-201 351 IWFCSEVESSTVTTRIVSHIKTKGALGVSFTFFGTSFLFITSHFTSGDGK 400

||||||||||||||||||||||||||||||||||||||||||||||||||

Affected 351 IWFCSEVESSTVTTRIVSHIKTKGALGVSFTFFGTSFLFITSHFTSGDGK 400

INPP5E-201 401 VSERLLDYSRTIQGLALPKSVPDTSPYRSDAADVTTRFDGVFWFGDFNFR 450

||||||||||||||||||||||||||||||||||||||||||||||||||

Affected 401 VSERLLDYSRTIQGLALPKSVPDTSPYRSDAADVTTRFDGVFWFGDFNFR 450

INPP5E-201 451 LSGGRVAVEAILKQDLVEKVSTLLQHDQLTQEMKKGSIFKGFQEPDIHFL 500

||||||||||||||||||||||||||||||||||||||||||||||||||

Affected 451 LSGGRVAVEAILKQDLVEKVSTLLQHDQLTQEMKKGSIFKGFQEPDIHFL 500

INPP5E-201 501 PSYKFDIGKDSYDTTSKQRTPS--YTDRVMYRSRHKGDICPVRYSSCPGI 548

||||||||.... |:.|. .:.:|.:...|: |:.|.|....||.

Affected 501 PSYKFDIGPGHV-----QKPPQGHLSSQVFFLPWHQ-DVPPPRVRPVPGQ 544

INPP5E-201 549 KTSDHR--PV---------------------------------YGLFRVK 563

..:..| |. |.|:||.

Affected 545 SEAGERQHPASCWQVPRAVLDRNQKTDFQRNPETASTEKSALEYDLYRVL 594

INPP5E-201 564 VRPGRDNIPLAAGKFDRELYLIGIKRRISKEIQRQQALKNQHSSTICTVS 613

Affected 595 -------------------------------------------------- 594
