# Supplementary material for: A splice site variant in INPP5E causes diffuse cystic renal dysplasia and hepatic fibrosis in dogs
Source: PLoS One. 2018 Sep 20;13(9):e0204073. doi: 10.1371/journal.pone.0204073 (PMC6147468; doi:10.1371/journal.pone.0204073)
Supplement: S1 Table — (DOCX) [file pone.0204073.s003.docx]

**S1 Table. Summary of exome sequencing and variant calling.**

| **Sample** | **Total number of reads** | **Reads mapped to CanFam3 (%)** | **Mean Coverage** | **Total variants /sample** | **Number of SNVs** | **Number of indels** |
| --- | --- | --- | --- | --- | --- | --- |
| CASE 3 | 61,730,566 | 60,092,578 (97,35%) | 36 | 211499 | 178291 | 32781 |
| CASE 2 | 74,523,972 | 72,004,569 (96,62%) | 38 | 217389 | 184247 | 32710 |
| Father of CASE 3 | 61,030,100 | 59,488,789 (97,47%) | 37 | 212123 | 172728 | 31562 |
| Mother of CASE 3 | 75,588,796 | 72,927,125 (96,48%) | 40 | 215763 | 183585 | 31786 |
| Control 1 | 71,426,490 | 68,929,042 (96,50%) | 39 | 210229 | 178766 | 31058 |
| Control 2 | 83,120,059 | 80,443,381 (96,78%) | 44 | 216974 | 184724 | 31848 |
| Control 3 | 80,583,218 | 77,876,628 (96,64%) | 42 | 218862 | 186291 | 32121 |
| Average | 72,571,886 | 70,251,730 (96,80%) | 39 | 214691 | 181233 | 31981 |
